# LPS-TLR4 Pathway Mediates Ductular Cell Expansion in Alcoholic Hepatitis

**DOI:** 10.1038/srep35610

**Published:** 2016-10-18

**Authors:** Gemma Odena, Jiegen Chen, Juan Jose Lozano, Jose Altamirano, Daniel Rodrigo-Torres, Silvia Affo, Oriol Morales-Ibanez, Hiroshi Matsushita, Jian Zou, Raluca Dumitru, Juan Caballeria, Pere Gines, Vicente Arroyo, Min You, Pierre-Emmanuel Rautou, Dominique Valla, Fulton Crews, Ekihiro Seki, Pau Sancho-Bru, Ramon Bataller

**Affiliations:** 1 Division of Gastroenterology and Hepatology, Departments of Medicine and Nutrition, University of North Carolina at Chapel Hill, Chapel Hill, NC, USA; 2 Institut d’Investigacions Biomèdiques August Pi i Sunyer (IDIBAPS), CIBER de Enfermedades Hepáticas y Digestivas (CIBERehd), Barcelona, Catalonia, Spain; 3 Division of Gastroenterology, Department of Medicine, Cedars-Sinai Medical Center, Los Angeles, CA, USA; 4 Bowles Center For Alcohol Studies, University of North Carolina at Chapel Hill, Chapel Hill, NC, USA; 5 UNC Human Pluripotent Stem Cell Core, Neuroscience Center and Department of Genetics, University of North Carolina at Chapel Hill, Chapel Hill, NC, USA; 6 Liver Unit, Hospital Clínic, Barcelona, Catalonia, Spain; 7 Department of Pharmaceutical Sciences, College of Pharmacy, Northeast Ohio Medical University, Rootstown, OH, USA; 8 Service d’Hépatologie, Hôpital Beaujon, Assistance Publique-Hôpitaux de Paris, Clichy, France; INSERM, U773, Centre de Recherche Biomédicale Bichat-Beaujon CRB3, Université Paris-Diderot-Paris 7, Hôpital Bichat, Paris, France; 9 INSERM, U970, Paris Cardiovascular Research Center-PARCC, and Université Paris Descartes, Sorbonne Paris Cité, UMR-S970, Paris, France

## Abstract

Alcoholic hepatitis (AH) is the most severe form of alcoholic liver disease for which there are no effective therapies. Patients with AH show impaired hepatocyte proliferation, expansion of inefficient ductular cells and high lipopolysaccharide (LPS) levels. It is unknown whether LPS mediates ductular cell expansion. We performed transcriptome studies and identified keratin 23 (KRT23) as a new ductular cell marker. KRT23 expression correlated with mortality and LPS serum levels. LPS-TLR4 pathway role in ductular cell expansion was assessed in human and mouse progenitor cells, liver slices and liver injured TLR4 KO mice. In AH patients, ductular cell expansion correlated with portal hypertension and collagen expression. Functional studies in ductular cells showed that KRT23 regulates collagen expression. These results support a role for LPS-TLR4 pathway in promoting ductular reaction in AH. Maneuvers aimed at decreasing LPS serum levels in AH patients could have beneficial effects by preventing ductular reaction development.

Alcoholic liver disease (ALD) is the main cause of cirrhosis worldwide^1^ and the main driver of health expenditure in hospitalized patients with liver disease in the US^2^. In clear contrast with the recent advances in viral hepatitis, there are no targeted therapies for patients with ALD. In particular, there are no effective therapies for alcoholic hepatitis (AH), a frequent and severe presentation of ALD patients that bears a high short-term mortality rate^3^. Mortality associated with AH is due to profound liver failure and portal hypertension, leading to complications such as variceal bleeding, renal failure and sepsis^4^,^5^. Patients with severe AH are particularly prone to bacterial infections, reflecting intense derangement of immune function^6^. The available therapy (*i.e.* prednisolone) does not improve survival beyond one month, and targeted therapies are urgently needed^7^,^8^,^9^. Identifying molecular and cellular drivers of AH is a prerequisite to develop such therapies. In fact, there is a current effort by public agencies in the US (i.e. NIH-sponsored international consortia on translational research in AH) to identify novel targets for therapy.

The pathogenesis of AH is largely unknown. Translational studies using human samples have identified several potential molecular targets including the CXC chemokine family, tumor necrosis factor receptor superfamily member 12A, osteopontin, chemokine (C-C motif) ligand 20, members of the inflammasome, interleukin-22, the Hedgehog signaling pathway and macrophage migration inhibitory factor^10^,^11^,^12^,^13^,^14^,^15^,^16^. Moreover, gut-derived bacterial products such as lipopolysaccharide (LPS) are believed to play a major role by inducing liver inflammation and fibrogenesis through toll-like receptor 4 (TLR4) expressed in both parenchymal and non-parenchymal cells^17^. Strategies aimed at modifying bacterial dysbiosis and ameliorating intestinal barrier dysfunction and the resulting translocation of endotoxin into the portal circulation may be beneficial in patients with AH^18^,^19^. We recently found that LPS serum levels predict mortality in patients with AH and are associated with a poor response to corticosteroids^20^. The mechanisms by which increased LPS levels are associated with a poor outcome are largely unknown. Human studies suggest that LPS could mediate immune paralysis in these patients and favor infections^21^,^22^. We hypothesize that LPS could also play a role in the impaired hepatocellular regeneration in these patients. Recent studies strongly suggest that an inefficient ductular reaction (mostly composed by liver progenitor cells -LPC-) could play a role in AH^23^,^24^. Furthermore, markers of hepatic ductular reaction at admission correlate with liver injury and closely predict short-term mortality in AH^23^ and patients non-responding to therapy show a massive expansion of ductular cells in the liver explants^24^. Little is known on the factors that regulate the growth and fate of ductular cells in the setting of AH. Investigating the biological properties of these cells could favor the development of novel targeted therapies for AH.

LPS is known to regulate the proliferation and fate in bone marrow, endothelial and dental progenitor cells through TLR4 signaling^25^,^26^,^27^,^28^,^29^. It is plausible that increased LPS levels also play a role in the expansion of inefficient ductular cells in AH. To test this hypothesis, we conducted a systems biology approach including a comparative transcriptome analysis of liver from patients with AH and non-alcoholic steatohepatitis (NASH) to find novel markers of ductular cells. The “structural molecule activity” pathway was found to be the most dysregulated pathway, and keratin 23 (KRT23) was the most upregulated gene in this family. Importantly, this keratin was expressed in the ductular reaction in humans and mice. Based on these recent data, we hypothesized that the LPS-TLR4 pathway may stimulate the expansion of ductular reaction and regulates the biological properties of ductular cells in AH.

## Results

### Identification of KRT23 as a Marker of Ductular Cells in AH

**Figure 1:**
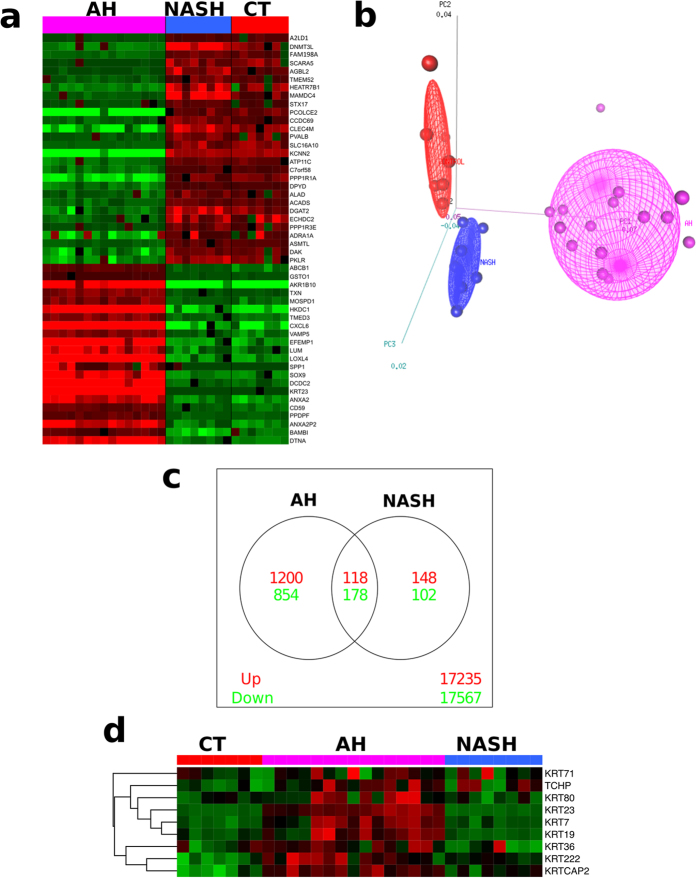
Comparative Functional Analysis of the Transcriptome in AH and NASH. (**a**) Heatmap display of the genes with the most significantly different expression between patients with AH, patients with NASH and healthy controls. Rows represent genes, and columns represent samples. The intensity of each color denotes the standardized ratio between each value and the average expression of each gene across all samples. Red pixels correspond to an increased abundance of mRNA in the indicated liver biopsy sample, whereas green pixels indicate decreased mRNA levels. (**b**) Multidimensional scaling analysis representing the 30 samples that underwent array profiling. The different samples are placed in the three-dimensional space according to their mRNA expression. AH samples are represented in magenta, NASH in light blue and normal livers in red. (**c**) Venn diagram showing the overlapping genes that were significantly upregulated (red) or downregulated (green). Venn diagram shows genes specifically upregulated (1200 genes) or downregulated (854 genes) in AH as well as genes specifically upregulated (148 genes) or downregulated (102 genes) in NASH. (**d**) Heatmap display of keratins with the most significantly different expression between AH and NASH and control livers. The intensity of each color denotes the standardized ratio between each value and the average expression of each gene across all samples. Red pixels correspond to an increased abundance of mRNA in the indicated liver biopsy sample, whereas green pixels indicate decreased mRNA levels.

Comparative gene expression profile analysis was performed in patients with severe AH (n = 15, Table 1), NASH (n = 8, Supplementary Table 1) and normal controls (n = 7) that underwent microarray analysis in our lab^11^,^30^ in order to identify genes specifically dysregulated in this severe form of ALD. Unsupervised clustering analysis revealed a unique gene expression signature of livers from patients with AH compared to NASH (Fig. 1a). To visualize the main differences in global transcriptional patterns between the three study groups we performed multidimensional scaling analysis of the 30 samples that underwent array profiling (Fig. 1b). Importantly, marked differences in gene expression were noted between livers from patients with AH, patients with NASH and normal controls (*P* < 0.001). A Venn diagram illustrates different groups of genes differentially regulated in both diseases (Fig. 1c). We found the majority of genes were differentially dysregulated between AH and NASH (1,200 upregulated and 854 downregulated in AH *vs.* NASH, respectively). Additionally, 296 genes were similarly dysregulated in AH and NASH compared to normal livers, while a smaller group of genes were specifically dysregulated in NASH compared to AH (148 upregulated and 102 downregulated, respectively). Functional analysis of the transcriptome data (GOstats) revealed key pathways differentially regulated in patients with AH when compared to NASH and control livers. Among these pathways, the “structural molecule activity” pathway was found to be the most significantly upregulated in AH (*P* = 7.5 × 10^−9^) (Supplementary Table 2). The most upregulated genes that were differentially regulated in this and other significant pathways between patients with AH and NASH are shown in Table 2.

Next, we sought to identify novel specific biomarkers exclusively overexpressed in patients with AH. We focused on the “structural molecule activity” pathway, the most dysregulated pathway in our functional analysis. Among the genes most upregulated in this pathway, we found several keratins (*KRT23*, keratin 19 *-KRT19*-, and keratin 7 -*KRT7*-) as well as genes encoding procollagens I-V (Table 2). Because keratins are novel biomarkers in liver disease that also regulate cell fate, we further analyzed this subgroup of genes. Figure 1c shows the expression of genes encoding keratins, which were markedly upregulated in AH. Among them, we found *KRT23* to be the most upregulated gene in AH compared to NASH and normal livers (45.8 and 126-fold increase, respectively, *P* < 0.001 for both).

**Figure 2:**
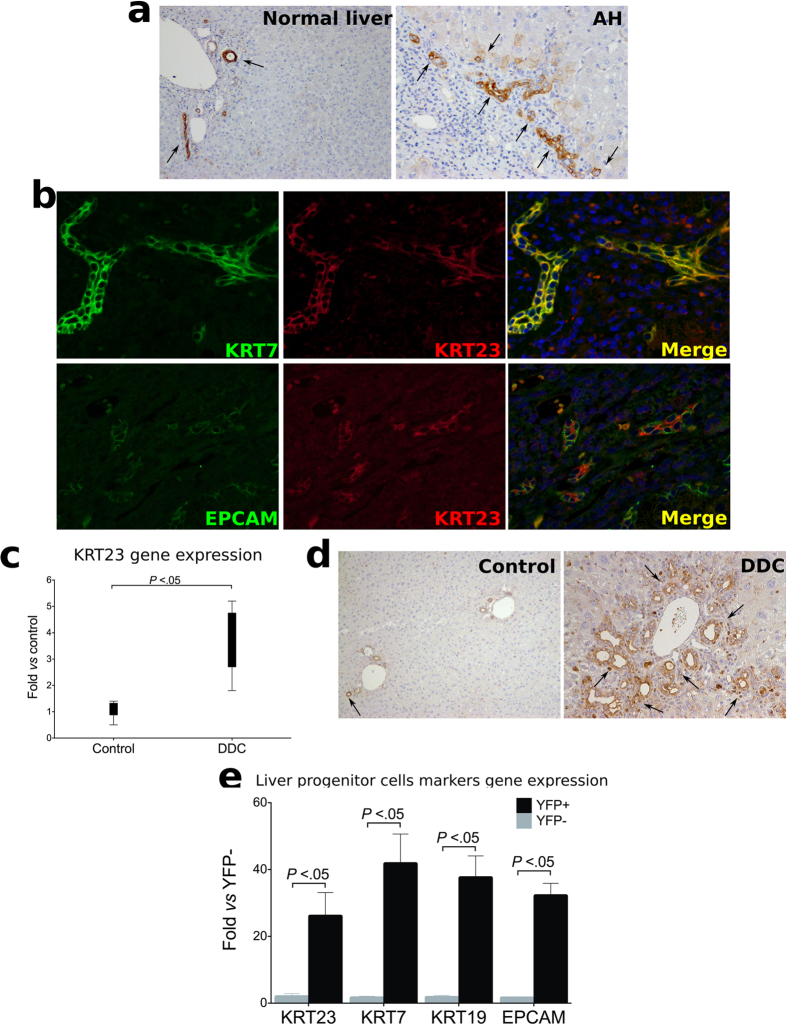
Identification of KRT23 as a Marker of Ductular Cells in AH. (**a**) Representative micrographs of KRT23 protein expression in paraffin sections of liver biopsies from normal livers and from patients with AH, stained with anti-KRT23 antibody (x100 magnification). (**b**) Immunofluorescence staining shows co-localization of KRT23 and LPCs markers (KRT7 and EPCAM) in paraffin sections of liver biopsies from patients with AH (x100 magnification). (**c**) Krt23 hepatic gene expression measured in a mouse model of progenitor cell expansion (DDC diet for 4 weeks, n = 6 compared to uninjured mouse liver (control, n = 6). (**d**) Representative images of liver KRT23 staining in mice treated for 4 weeks with a DDC diet and in uninjured mice (x200 magnification). (**e**) *Krt23* and LPCs markers (*Epcam*, *Krt7*, *Krt19*) gene expression was significantly enriched in LCP cells (n = 3) *vs.* a non-LPC population (n = 3).

Based on previous studies from our lab showing that most upregulated genes in AH are ductular reaction markers^23^, we hypothesized that KRT23 is predominantly expressed in these cells. Immunohistochemistry studies in patients with AH, including double staining studies using two ductular reaction markers (KRT7, epithelial cell adhesion molecule -EPCAM-) clearly demonstrated that KRT23 is preferentially expressed in the ductular reaction cells in AH (Fig. 2a,b). KRT23 showed a strong overlap with KRT7 and co-localized in the same cell as EPCAM. KRT23 is also weakly expressed in some hepatoblasts in the vicinity of the ductular reaction. These results strongly suggest that KRT23 is mainly expressed in the ductular reaction (likely LPCs) in patients with AH.

### Expression of KRT23 in Animal Models of Ductular Reaction

The current experimental models of ALD do not reproduce the main histological features of severe AH (*i.e.* advanced fibrosis, bilirrubinostasis and ductular reaction) that are associated with a poor outcome^23^,^31^,^32^ but rather mimic moderate alcoholic liver disease. To overcome this difficulty, we used different animal models of liver injury which reproduce unique features of AH. To explore the expression of KRT23 in the ductular reaction we used a well-characterized experimental model which causes advanced fibrosis, cholestasis and progenitor cell expansion (*i.e.* DDC diet). We found that KRT23 gene and protein expression were dramatically induced in DDC-treated mice. Interestingly, KRT23 protein was detected within the ductular reaction (Fig. 2c,d). To further confirm that KRT23 is expressed in LPCs, we analyzed hepatic gene expression of primary LPCs isolated from DDC-treated mice^33^. Expression of KRT23 as well as specific cell markers of LPCs were significantly enriched in LPC cells *vs.* non-LPC population (Fig. 2e). Overall, these results indicate that KRT23 is expressed in the ductular reaction and LPCs in livers with cholestasis and progenitor cell expansion.

### Expression of KRT23-positive Ductular Cells in AH: Correlation with Disease Outcome

**Figure 3:**
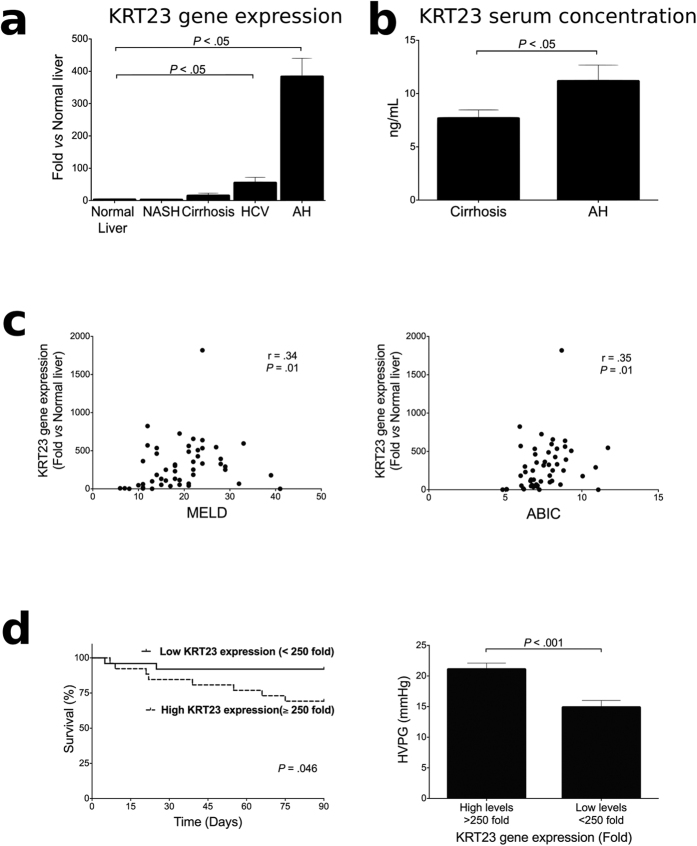
KRT23 expression correlates with disease severity and key features of AH. (**a**) *KRT23* hepatic gene expression measured by qPCR in patients with AH (n = 51), HCV (n = 10), compensated cirrhosis (n = 10) and NASH (n = 14), compared to normal livers (n = 7). (**b**) KRT23 peripheral serum levels are elevated in patients with AH (n = 34) when compared to patients with cirrhosis (n = 14). (**c**) Correlation between *KRT23* hepatic gene expression (fold change vs. normal livers) and clinical features in patients with AH (n = 51). Both MELD score as well as ABIC score correlated with *KRT23* hepatic gene expression. (**d**) Kaplan-Meier’s curve analysis illustrates the relationship between *KRT23* hepatic gene expression with 90-day mortality in patients with AH. A cut-off value of 250-fold expression (fold change vs. normal livers) defined patients with low and high *KRT23* gene expression with the best sensitivity and specificity. Portal hypertension (hepatic venous pressure gradient – HVPG – mmHg) was higher in patients with higher *KRT23* gene expression levels (>250-fold).

To confirm the microarray results, we analyzed KRT23 hepatic gene expression in a large cohort of patients with AH and other diseased controls by real time PCR. The results confirmed a dramatic upregulation of *KRT23* in patients with AH (n = 51) compared with normal livers (n = 7) and other liver diseases including compensated cirrhosis (n = 10), hepatitis C (n = 10, HCV) and NASH (n = 14), (*P* < 0.001) (Fig. 3a). *KRT23* expression was also upregulated, but to a lesser extent, in patients with HCV (*P* < 0.005) and compensated cirrhosis (*P* < 0.001) compared to the control liver samples. Elevated circulating levels of soluble keratin 18 have been detected in several liver diseases and may serve as a biomarker for identifying steatohepatitis^34^,^35^. We then assessed serum KRT23 concentration and plasma free and microparticle-bound proteins by ELISA as a potential biomarker of liver disease. KRT23 serum concentration was higher in patients with AH as compared to patients with compensated cirrhosis (Fig. 3b). Since other keratins (*i.e.* keratin 18) are typically bound to microparticles, we next explored whether KRT23 is also linked to these small molecules. We found that the vast majority of KRT23 in plasma was not bound to microparticles (Supplementary Fig. 1a,b).

To gain insight on the pathogenic role of KRT23-positive ductular cells in AH, we next explored whether its expression correlated with disease severity in the cohort of patients with AH (n = 51). KRT23 hepatic gene expression (expressed as fold change vs. normal livers) positively correlated with the main prognostic scores in patients with AH. Hepatic *KRT23* correlated with Model for End-stage Liver Disease (MELD; *P* = 0.01) and Age/Bilirubin/International normalized ratio/Creatinine (ABIC; *P* = 0.01) scores (Fig. 3c). We also observed increased hepatic *KRT23* mRNA levels in patients who died within 90 days after admission compared with those who survived (522-fold *vs.* 245-fold induction, respectively; *P* = 0.01). In addition, to evaluate if *KRT23* can serve as a predictor of short-term mortality, we performed a Kaplan-Meier analysis. A value of 250-fold expression (fold expression vs. normal livers) was identified as the cut-off value with better sensitivity and specificity to define patients with low and high *KRT23* gene expression (area under the receiver characteristic curve - AUROC = 0.72 [95% CI 0.51–0.91]). *KRT23* hepatic gene expression predicted short-term mortality in patients with AH (Fig. 3d). Finally, we analyzed if *KRT23* gene expression correlated with the degree of portal hypertension, a major pathophysiological event in these patients. We observed a higher degree of portal hypertension in patients with higher levels of hepatic *KRT23* expression compared to those with lower levels of hepatic *KRT23* expression (hepatic venous pressure gradient 14 ± 6 mmHg *vs.* 21 ± 5 mmHg, respectively; *P* < 0.001) (Fig. 3d). Overall, these results suggest that KRT23-positive ductular cells may play a role in the pathophysiology of AH and that KRT23 might be used as a biomarker to predict short-term outcomes in patients with AH.

### Role of LPS-TLR4 Pathway on Expression of KRT23-positive Ductular Cells

**Figure 4:**
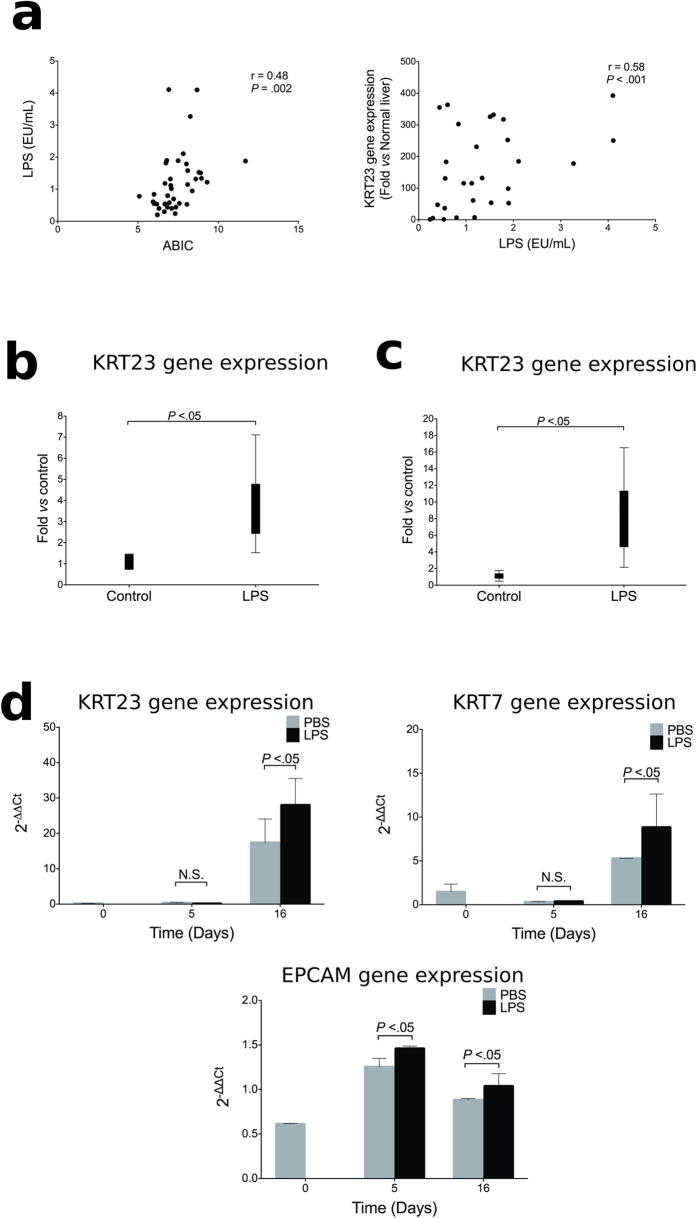
KRT23 hepatic expression in animal models of liver injury and in LPCs. (**a**) Relationship between *KRT23* hepatic gene expression and LPS serum levels in patients with AH (n = 39). Correlation between LPS serum levels and ABIC score in patients with AH. (**b**) *Krt23* hepatic gene expression in a mouse model of acute liver injury. *Krt23* gene expression in mice treated with LPS (n = 6) compared to control mice (n = 6). (**c**) *Krt23* hepatic gene expression in a mouse model of advanced fibrosis. *Krt23* in mice treated CCl_4_ plus LPS (n = 8) compared to mice treated with CCl_4_ alone (n = 6). (**d**) Gene expression of *KRT23* and LPCs markers (*KRT7* and *EPCAM*) in a model of liver progenitor cell differentiation from 3 independent experiments.

LPS plays a major role in several liver diseases^36^,^37^,^38^,^39^. Because LPS is a major driver in AH and correlates with patient outcome^20^, we next explored if LPS stimulates the expression of ductular cell makers using different experimental approaches. First, we showed that LPS serum levels strongly correlated with hepatic expression of *KRT23* in patients with AH (*P* < 0.001, Fig. 4a). To uncover the mechanisms driving the increase of KRT23-positive ductular cells in AH, we used different animal models of liver injury which are representative of some of the key events that occur in AH, such as ethanol consumption, fibrosis and endotoxaemia. First, we showed that LPS administration markedly induced *Krt23* expression in Balb/c mice (Fig. 4b). Then, we explored if LPS further increases the expression of *Krt23* in livers with advanced fibrosis. To test this hypothesis we induced advanced fibrosis with chronic carbon tetrachloride (CCl_4_). As shown in Fig. 4c LPS administration further increased the expression for *Krt23* in fibrotic livers. These results were confirmed in precision-cut liver slices (Supplementary Fig. 2a–h). Finally, we used a protocol to generate LPCs from pluripotent stem cells. Differentiation of these stem cells into LPCs resulted in the upregulation of keratins including KRT23 and KRT7 as well as EPCAM, a ductular reaction marker. Notably, expression of these markers was increased in cells incubated with LPS (Fig. 4d and Supplementary Fig. 3a–c). These results, together with the finding that LPS serum levels correlated with KRT23 expression in AH, strongly suggest that LPS could drive expression of KRT23-positive ductular cells.

**Figure 5:**
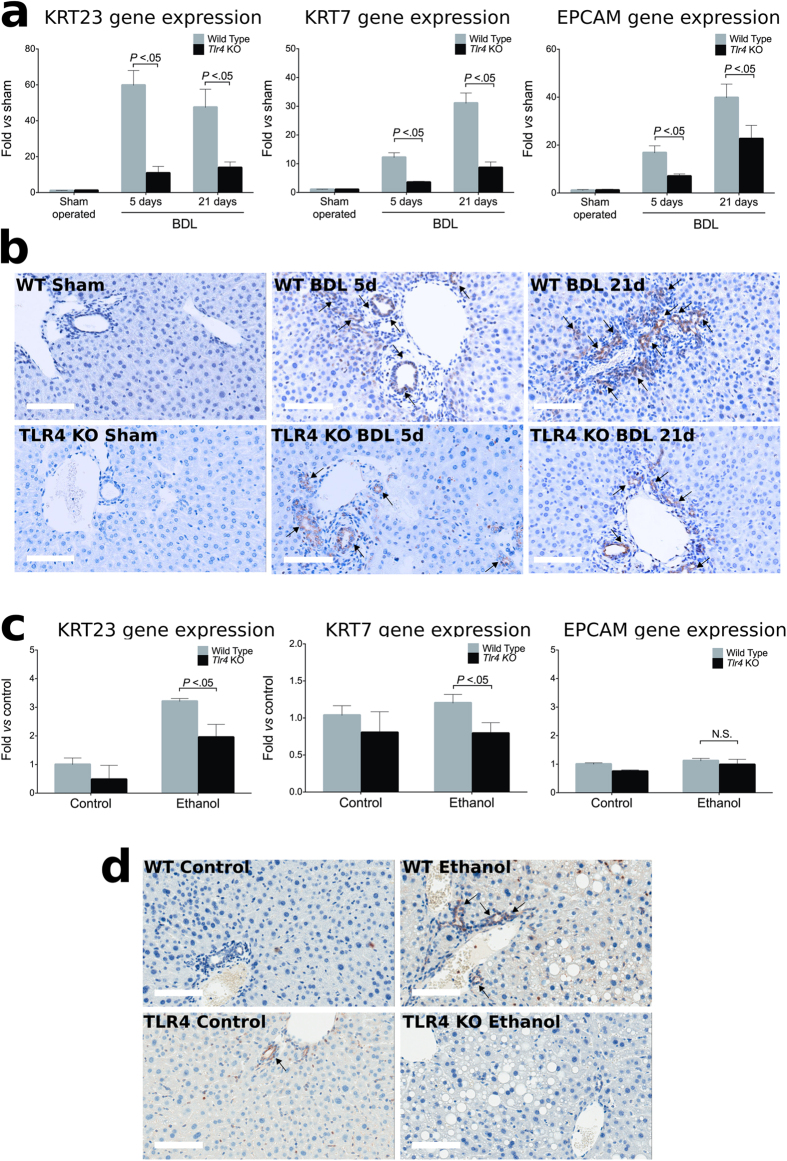
LPS-TLR4 induced liver damage is mediated by ductular cells in animal models of liver injury. (**a**) Gene expression of *Krt23* and LPCs markers (*Krt7* and *Epcam*) in WT and *Tlr4*-KO mice subjected to sham operation (n = 3) or BDL. BDL mice were sacrificed 5 (n = 4) or 21 days (n = 8) after operation. (**b**) Representative micrographs of KRT23 protein expression in WT and *Tlr4*-KO mice subjected to sham operation or BDL. (**c**) Gene expression of *Krt23* and LPCs markers in WT and *Tlr4*-KO mice subjected to Tsukamoto-French model of ethanol damage (4 weeks, n = 6) compared to uninjured control mice (n = 4). (**d**) Representative micrographs of Krt23 protein expression in WT and *Tlr4*-KO mice subjected to Tsukamoto-French model and uninjured control mice. Bars, 100 μm.

Subsequently, we investigated whether TLR4 mediates the effect of LPS on the development of KRT23-positive ductular reaction. To test this hypothesis, we first found that TLR4 small interfering RNA (*Tlr4* siRNA) blocked LPS-induced *Krt23* in precision-cut liver slices (Supplementary Fig. 2a,b). Next, we performed *in vivo* loss-of-function studies using *Tlr4* deficient mice. We first caused advanced fibrosis by inducing chronic cholestasis through prolonged bile duct ligation (BDL), which is known to increase serum LPS levels^40^. Mice with BDL showed a marked upregulation of KRT23 (both at the gene and protein levels), KRT7, and EPCAM, all of which were heavily blunted in mice lacking TLR4 (Fig. 5a,b). To a lesser extent, these results were also observed in TLR4-KO mice chronically exposed to alcohol (Tsukamoto-French model) (Fig. 5c,d). Overall, these results suggest that the LPS-TLR4 pathway mediates the development of ductular reaction and KRT23 expression in chronic liver injury.

### Regulation of KRT23 Expression by Histone Acetylation in LPCs

**Figure 6:**
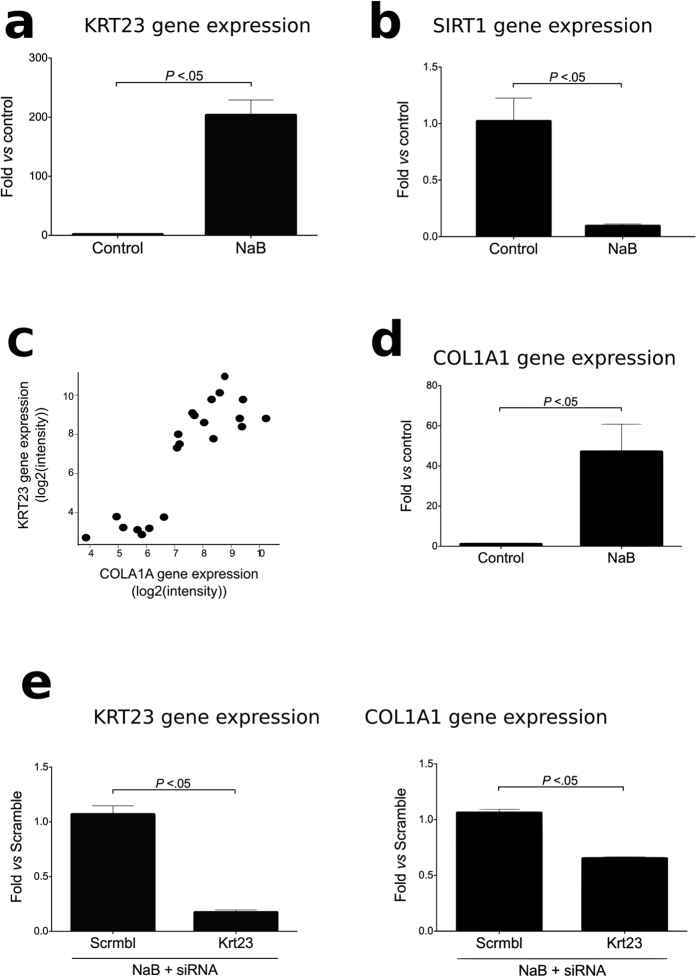
KRT23 regulation by HDACs in mice liver progenitor cells and KRT23 effects on collagen synthesis. (**a**) Effect of HDAC inhibition by NaB administration on *Krt23* gene expression in BAML cells (3 independent experiments). (**b**) Gene expression of *Sirt1*, an HDAC, in a BAML cells treated with NaB (3 independent experiments). (**c**) Correlation between *KRT23* hepatic gene expression and *COL1A1* hepatic gene expression in patients with AH (n = 39). (**d**) Effect of HDACs inhibition by NaB administration on *Col1a1* gene expression in BAML cells. (**e**) Silencing of *Krt23* by means of siRNA inhibited NaB induced *Col1a1* gene expression in BAML cells (3 independent experiments).

Because ALD is characterized by profound epigenetic changes^41^ and KRT23 is sensitive to histone deacetylases (HDAC) inhibition^42^, we next investigated whether KRT23 expression in ductular cells is governed by histone acetylation in LPCs. First, we showed that an exogenous inhibitor of HDACs (sodium butyrate -NaB-) considerably increased *Krt23* in bipotential mouse adult liver cells (BAML) isolated from mice (Fig. 6a). We recently demonstrated that AH is characterized by a distinct decrease of sirtuin 1 (SIRT1), a powerful HDAC^43^. To test if SIRT1 also plays a role in KRT23 induction, we first investigated if NaB treatment on BAML cells is associated with a decrease in SIRT1 levels. As shown in Fig. 6b, *SIRT1* levels are downregulated in this model. *SIRT1* levels are also downregulated in the model of human LPC differentiation in culture (Supplementary Fig. 6a,b). The role of SIRT1 in inducing KRT23 expression was demonstrated in mice deficient for *Sirt1* exposed to chronic ethanol (Supplementary Fig. 5a,b). The absence of *Sirt1* in mice exposed to alcohol was associated with increased Krt23 expression. These results suggest that histone acetylation regulates KRT23 expression in LPCs.

### Fibrogenic Roles of KRT23 in Ductular Cells

Because severe fibrosis is a hallmark find in AH that predicts survival in these patients^31^, and levels of KRT23 correlated with the degree of portal hypertension in patients with AH, we finally explored the potential fibrogenic role of KRT23 in ductular cells. We found that the expression of *KRT23* closely correlated with the expression of collagen type I alpha 1 (*COL1A1*) (Fig. 6c). Since ductular cells express COL1A1, we next investigated if KRT23 stimulates profibrogenic actions in mouse BAML cells. BAML cells treated with NaB also showed *Col1a1* over-expression in a *Krt23*-dependent manner (Fig. 6d), since this profibrogenic response was blunted in cells transfected with a *Krt23* siRNA (Fig. 6e). Taken together, these results suggest that KRT23 may be promoting pro-fibrogenic actions in ductular cells.

## Discussion

The current paradigm is that AH is due to a flare of hepatic inflammation caused by neutrophils in a previously damaged liver. However, we recently found that neutrophil infiltrate is associated with a better prognosis in AH^31^ and that severe AH is characterized by poor hepatocellular function and an inefficient accumulation of ductular cells^23^,^24^. Therefore, our efforts are currently focused in finding the molecular drivers causing this massive accumulation of ductular cells. To identify new markers of ductular cells, we used a highly translational approach. We first compared the transcriptome of patients with AH and NASH, and found that the “structural molecule activity” pathway was the most specifically dysregulated in patients with AH. A detailed analysis of this family of genes allowed us to identify several keratins that were profoundly upregulated in the livers with AH. Among them, we found that *KRT23* was by far the most upregulated gene in this family. Interestingly, KRT23 has been previously reported as a biomarker for steatohepatitis^44^. In the Starmann *et al*. study, patients with steatohepatitis presented an advance degree of fibrosis. Moreover, 5 out of 8 patients with steatohepatitis included in their microarray cohort presented ALD. These are common traits between Starmann *et al*. and the AH cohort of the present study. Thus, KRT23 may be expressed in cases of steatohepatitis with cirrhosis.

Because many of the most overexpressed genes in AH are mainly expressed in ductular cell/LPCs, we hypothesized that KRT23 could mainly be expressed in these cells. Our further histological studies in human samples and experimental approaches in animal models of ductular reaction strongly suggest that this keratin is mainly expressed in the ductular reaction/LPCs population. Our results are in accordance with a recent study from Guldiken *et al*. showing that KRT23 is a maker of mouse and human ductular reaction^45^. Thus, we used KRT23 together with other known markers of LPCs (KRT7 and EPCAM) to study the mechanisms leading to the massive accumulation of ductular cells in livers with severe AH.

It is well known that proliferative bile ductular reaction not only occurs in AH but also in various liver diseases including acute-on-chronic liver failure and advanced ALD^46^. Whether the ductular reaction contributes to liver regeneration is still unclear as experimental studies show contradictory results^47^,^48^. Using genetic lineage-tracing, we previously showed that the biliary compartment probably does not play a role during normal liver homeostasis. However, following profound liver injury, biliary cells give rise to an expanding LPC population that have the potential to generate hepatocytes^33^. Yet, the contribution of the ductular reaction in the regeneration of the failing liver in AH is probably minimal. This assertion is based on two previous observations from our research groups. First, the accumulation of ductular/LPCs in patients with AH, assessed as the expression of specific cells markers, closely predicts patient mortality^23^. In other words, the more ductular cells that accumulate in the liver, the worse the patient’s short-term prognosis is. Second, explants from patients that did not respond to corticosteroids showed a massive accumulation of ductular cells together with poor hepatocellular function and proliferation^24^. Importantly, in these patients with burn-out AH, bipotent LPCs are mainly engaged to differentiate into a biliary phenotype, rather than into hepatocytes. Altogether, these results strongly suggest that the ductular reaction does not contribute to the regeneration of dying hepatocytes. On the contrary, it is plausible that accumulation of these cells produces deleterious effects such as lobular fibrosis. Our results show that LPCs express collagen and support this hypothesis. Interestingly, our immunohistochemistry study in patients with AH revealed that some hepatoblasts in the vicinity of the ductular reaction weakly express KRT23. KRT23 expression in hepatoblasts is also suggested in Guldiken *et al*.^45^. This finding suggests that cells derived from LPCs progressively lose this marker. However, it is also possible that in the context of AH, stressed hepatocytes undergo reprogramming and *de novo* express keratin including KRT23. Further studies should test this intriguing hypothesis and should evaluate the gene expression profile and biological actions of LPCs within the ductular reaction in non-alcoholic forms of acute-on-chronic liver failure.

An important finding of our study is that ductular reaction and the consequent KRT23 expression are driven by LPS/TLR4. This finding suggests that increased LPS in patients with severe forms of AH could promote ductular reaction and keratin expression. The fact that LPS levels correlate with *KRT23* gene expression and that mice lacking *Tlr4* have attenuated ductular reaction and keratin expression (Krt23 and Krt7) support this hypothesis. Moreover, IL-1ß induces *Krt23* gene expression in hepatocellular and bile duct cells lines suggesting an association with inflammatory reaction^45^. There is mounting evidence that LPS plays multiple pathogenic roles in AH and is a major driver in this condition^21^. First, LPS serum levels measured at admission predict multiple organ failure, mortality and corticosteroids response in patients with AH^20^. Second, LPS serum levels correlate with neutrophil dysfunction and survival in AH, suggesting that the LPS-TLRs pathway plays a major role in immune paralysis in this population^49^. Third, TLRs mediate the release of inflammatory and fibrogenic mediators from immune and non-parenchymal cells caused by gut-derived endotoxin^50^. All together, these studies indicate that targeting LPS translocation from the gut represents an appealing strategy to treat patients with AH.

We also explored other mechanisms regulating KRT23 expression. KRT23 expression is known to be sensitive to histone deacetylase inhibition^42^. We demonstrated that *KRT23* gene expression is strongly induced in LPCs exposed to exogenous inhibitors of HDACs. To explore if endogenous HDACs also play a role on *KRT23* expression, we focused on sirtuin 1, which we recently showed to be downregulated in patients with AH^43^. Interestingly, *KRT23* induction correlated with decreased *SIRT1* in a cell model of differentiation of LPCs from stem cells, and Krt23 protein expression was increased in *Sirt1*-KO mice exposed to chronic ethanol. Collectively, our results suggest that histone acetylation regulates KRT23 expression in ductular cells. Further studies are needed to investigate the regulation of ductular KRT23-positive cells through histone acetylation in AH.

Finally, we explored whether KRT23 regulates the biological functions of ductular cells/LPCs. As stated above, in patients with AH, cells within the ductular reaction have a highly proliferative phenotype that is probably linked to poor differentiation into mature hepatocytes^24^. Moreover, AH is characterized by pericellular and sinusoidal fibrosis, and we previously demonstrated that the stage of fibrosis is significantly associated with 90-day survival in these patients^31^. Therefore, we explored the potential role of LPS-TLR4-KRT23 on collagen expression of LPCs. We found that *KRT23* regulates the expression of *COL1A1* in ductular cells. In fact, several studies indicate that the inhibition of the LPC response to liver injury is associated with a reduction in the severity of hepatic fibrosis^51^,^52^. The role of collagen production by ductular reaction cells in AH needs to be further explored, as myofibrobalsts are probably the main fibrogenic cell type in liver diseases regardless of the origin. Further studies should investigate whether targeting these actions in LPCs has beneficial effects in AH.

Our study has potential limitations. First, the exact contribution of ductular cells to liver regeneration in the setting of AH is largely unknown. Second, there are no well-established animal models of AH that mimic the main findings in humans (i.e. severe fibrosis and cholestasis), since available models only show signs of mild to moderate steatohepatitis. To overcome these limitations, we are currently optimizing the isolation procedure of single cells from biopsies form patients with AH. Single-cells studies will allow us to further characterize the origin and biological properties of ductular cells.

In summary, the present study provides evidence that KRT23, one of the most upregulated genes in AH, is a novel marker of ductular cells and correlates with disease severity. Moreover, we found that the LPS-TLR4 pathway drives accumulation KRT23-positive ductular cells, and that KRT23 which regulates their pro-fibrogenic functions *in vitro*. Further preclinical studies in models of true AH are required to determine if targeting KRT23-positive cells is an effective and safe therapeutic strategy to reinstate differentiation of these cells into mature hepatocytes and improve liver function.

## Methods

### Patients

Patients admitted to the Liver Unit of the Hospital Clínic, Barcelona, between January 2007 and December 2009 with clinical, analytical and histological features of AH were included in a prospective fashion. Experiments were performed with approval and under the accordance of the relevant guidelines established by The Ethics Committee of the Hospital Clinic of Barcelona. All patients included gave written informed consent. The inclusion criteria have been previously described^10^,^53^,^54^. Microarray data were deposited in NCBI’s Gene Expression Omnibus (GEO; accession number GSE28619). Patients with malignancies or any other potential cause of liver disease were excluded from the study. Liver biopsies were obtained using a transjugular approach. A fragment of liver tissue, serum and plasma were obtained at the time biopsy. A total of 51 patients with AH were included (Table 1). As diseased controls, we included liver tissue from treatment-naïve chronic hepatitis C virus (HCV) infection (genotype 1) (n = 10), compensated alcoholic cirrhosis patients without active alcohol intake for at least 6 months (n = 10) as well as patients with morbid obesity and NASH (n = 14) according to Kleiner’s criteria^55^. Only patients with NASH without evidence of excessive alcohol intake (>20 gr/d) or other potential cause of liver disease were included (Supplementary Table 1). Additionally, as normal controls we included fragments of normal livers obtained from optimal cadaveric liver donors (n = 3) or resection of liver metastases (n = 4). Criteria for selected normal livers have been described in detail previously^11^.

### Comparative Microarray Studies

Comparative analysis of microarray data from patients with AH and NASH was performed. Whole transcriptome analysis from these two types of patients was previously performed by our group^11^,^30^. All microarray studies were performed at the same time using Human Genome U133A plus 2.0 GeneChip (Affymetrix Hgu133plus, Santa Clara, CA). Differential expression was assessed by using linear models and empirical Bayes moderated t-statistics. Linear Models for Microarray Analysis (LIMMA) R-package software was used for the analysis of gene expression microarray data^56^. Group comparisons and determinations of false discovery rates (FDR computation using Benjamini-Hochberg procedure)^57^ were performed. Functional analysis of gene expression data was conducted using the R/Bioconductor package GOstats and the GO database (http://www.geneontology.org). Only genes that could be associated with a unique EntrezGene ID were used. Among them those were selected representing a change of 1.5-fold or greater and moderated p-value < 0.05. The hypergeometric distribution was used to evaluate the probability of randomly observing the enrichment for each GO term^58^.

### Experimental Models of Liver Injury and Progenitor Cell Expansion in Mice

The current experimental models of ALD do not reproduce the main histological features of severe AH but rather mimic moderate alcoholic liver disease^32^. Through the study we used different animal models to better mimic particular features of AH. All mice used were male.

To investigate the effects of the LPS-TLR4 pathway in KRT23 induction we used a model of acute liver injury (LPS) and advanced fibrosis (CCl_4_ followed by a single dose of LPS)^11^. Male 8-week-old Balb/c mice were purchased from Charles River (Charles River, l’Arbresle, France). For the acute liver injury model mice were injected LPS (Sigma-Aldrich) intravenously at a dose of 10 mg/kg body weight (n = 6) or saline as control (n = 6) and were sacrificed 4 hours after the injection. For the advanced fibrosis model, mice were injected carbon tetrachloride (CCl_4_) intraperitoneally (diluted 1:4 in corn oil; Sigma-Aldrich, St Louis, MO) at a dose of 0.5 ml/kg body weight twice per week for a total of 5 injections. Control mice (n = 6) were given vehicle (corn oil, Sigma-Aldrich). CCl_4_ treated mice were injected intravenously either LPS at a dose of 10 mg/kg body weight (n = 8) or saline as control (n = 6) and were sacrificed 4 hours after the injection.

A model of ductular reaction expansion was induced by administering a 0.1% 3,5-diethoxycarbonyl-1,4-dihydro-collidin (DDC) diet for 3 weeks (n = 6). LPCs and non-LPCs cells were isolated by liver perfusion method from Hnf1βCreER/R26RYfp uninjured and from mice treated with a DDC containing diet (Sigma-Aldrich) for 3 weeks as previously described^33^.

Pathogen-free 8-week-old male TLR4-KO mice (originally generated by Dr. Shizuo Akira, Osaka University, Japan), and wild-type (WT) littermates were subjected to a model of ductular reaction (BDL) or to a model of alcoholic steatohepatitis (Tsukamoto-French)^59^. Briefly, the murine TLR4 genomic clone was screened from the 129/SvJ mouse genomic library (Stratagene, La Jolla, CA). A targeting vector was designed and electroporated into E14.1 embryonic stem cells. Two embryonic stem cell clones were selected for microinjection into blastocysts. These clones gave germline transmission and were interbred to generate homologous gene targeted mice. TLR4 knowckout mice were backcrossed at least 10 generations onto the C57BL/6 background. Bile duct ligation (BDL) was performed as described previously^50^,^60^. Bile duct ligated and sham-operated mice (n = 3) were sacrificed 5 (n = 4) or 21 (n = 8) days after surgery. Tsukamoto-French model of continuous ethanol infusion in mice was performed in as described previously^50^,^61^. Ethanol infusion was increased until it reached 29.4 g/kg/day at 4 weeks (n = 6; control mice n = 4).

A model of chronic plus single binge ethanol in *Sirt1*-KO mice was used to explore the role of HDACs as modulators of KRT23 expression. Liver-specific sirtuin KO mice (*Sirt1*-KO) (n = 6) mice and their age-matched WT littermates (n = 6) were subjected to a model of chronic plus single binge ethanol consumption as previously described (NIAAA model)^43^. *Sirt1* allele with floxed exon 4 was backcrossed 5 times into the C57BL/6 background. It was then bred with mice expressing the Cre recombinase driven by the albumin promoter to generate liver specific *Sirt1* knockout mice (S*irt1* LKO) in over 98% C57BL/6 background. SIRT1 LKO mice and their age-matched littermate Lox controls (*Cre*^−/−^, *Sirt1*^flox/flox^) older than 6-week of age were used in the present study. All animal procedures were approved by The University of North Carolina at Chapel Hill Institutional Animal Care and Use Committee and were conducted in accordance with the National Institutes of Health Guide for the Care and Use of Laboratory Animals.

### Cell cultures and *in vitro* assays

To investigate the effect of LPS on ductular cells, the NIH-approved human pluripotent stem cells line H1 (WA01, WiCell Research Institute, Wisconsin, MA) underwent differentiation following The National Institutes of Health Guidelines on Human Stem Cell Research, as described previously^62^. Briefly, differentiation of H1 PSCs cells was induced by culturing the cells in priming medium (media formulation described in Supplementary methods). After 72 hours, differentiation medium was added for 5 days. At day 8, cells were cultured in maturation and maintenance medium for 8 days. Cells were collected for RNA and protein extraction as well as immunofluorescense analysis after 5 days in differentiation medium and after 8 days in maturation medium. Thirty-six hours before harvest, cells were treated with LPS (1 μg/mL, Sigma-Aldrich) or phosphate buffered saline (PBS, Sigma-Aldrich).

BAML cells were isolated from control mice as previously described^63^ and were maintained on type-I collagen-coated plates in MCDB201/DMEM medium. BAML cells were treated with the HDAC inhibitor sodium butyrate (NaB, 4 mM, Sigma-Aldrich) for 24 h in fresh culture medium. Transfection with either scrambled or KRT23 small interfering RNA (siRNA; Life Technologies) was performed for 24 h and followed by treatment with 4 mM NaB for 24 h in fresh culture medium. Lipofectamine^®^ 3000 reagent (Life Technologies) was used according to the manufacturer’s instructions. Transduction experiments with adenovirus vectors containing either a specific human KRT23 sequence (Ad-*KRT23*) or a GFP sequence (Ad-*GFP*)(SignaGen Laboratories, Rockville, MD) incubated in cell culture media for 12 h were performed on human HepaRG cells (Life Technologies).

### Analysis of Serum KRT23 levels

Samples from patients with AH (n = 37) and compensated alcoholic cirrhosis (n = 14) were analyzed. To detect KRT23 serum levels, serum samples were collected from patients and stored at −80 °C. Serum samples were analyzed using commercially available immunoassays for KRT23 detection (CSB-EL012539HU ELISA kit; Cusabio, Wuhan, China). LPS serum levels were determined using the limulus amoebocyte lysate QCL-1000 test (Lonza Walkersville Inc, Walkersville, MA).

### Statistics

Continuous variables were described as means (±standard error) or medians (interquartile range). Comparisons between groups were performed using the Student’s *t* test or the Mann-Whitney *U* test, depending on their normality test. Differences between categorical variables were assessed by Fisher’s exact test or the chi-square test with Yates correction for continuity, when necessary. Correlations between variables were evaluated using Spearman’s *rho* or Pearson’s *r*, when appropriate. The area under the receiver characteristic curve (AUROC) analysis was used to determine the best cut-off value and the accuracy (sensitivity and specificity) of continuous variables associated with 90-day mortality. A comparative risk analysis was performed using the Kaplan-Meier method. Comparisons were performed by the log-rank test. All statistical analyses were performed using SPSS version 14.0 for Windows (SPSS Inc., Chicago, IL).

## Additional Information

**How to cite this article**: Odena, G. *et al*. LPS-TLR4 Pathway Mediates Ductular Cell Expansion in Alcoholic Hepatitis. *Sci. Rep.* **6**, 35610; doi: 10.1038/srep35610 (2016).

## Supplementary Material

Supplementary Information

## Figures and Tables

**Table 1:** Baseline characteristics of patients with AH.

Characteristics	Median (25–75 IQR)
Age (years)	52 (46–56)
Male n (%)	39 (76)
Corticosteroids n (%)	27 (53)
SIRS (%)	18 (35)
Laboratory and hemodynamic parameters	
Hemoglobin (g/dL)	17 (10–13)
Leukocyte count x10^9^/L	8.6 (6.3–12.6)
Platelet count x10^9^/L	119 (77–208)
AST (U/L)	117 (66–152)
ALT (U/L)	37 (23–60)
Serum albumin (g/dL)	2.6 (2.3–3.2)
Serum creatinine (mg/dL)	0.9 (0.6–1.1)
Serum bilirubin (mg/dL)	6.7 (3.0–18.5)
International normalized ratio	1.6 (1.4–1.8)
Hepatic venous pressure gradient (mmHg)	19 (14–22)
Alcoholic hepatitis severity scores at admission	
MELD score	19 (14–24)
ABIC score	7.3 (6.7–8.4)
ABIC Class n (%)	
A	14 (27)
B	31 (61)
C	6 (12)
Clinical decompensations during hospitalization	
AKI n (%)	18 (35)
Infection n (%)	17 (33)
Mortality at 90 days n (%)	10 (20)

Note: The total series included 51 patients. Samples from 15 patients were used for microarray analysis. IQR, interquartile range; SIRS, Systemic inflammatory response syndrome; AKI, Acute kidney injury.

**Table 2:** Most relevant genes differentially expressed in patients with AH compared with patients with NASH.

Accession N°	Gene name or Symbol	Fold Change	Accession N°	Gene name or Symbol	Fold Change
Structural molecule activity			Identical protein binding		
NM_015515	Keratin 23	45.8	NM_005564	Lipocalin 2	11.1
NM_000089	Collagen, type I, α 2	10.1	NM_178859	*SLC51B*	6.3
K01228	Collagen, type I, α 1	8.1	AF133207	*HSPB8*	4.9
NM_002276	Keratin 19	7.1	NM_001673	*ASNS*	4.5
BC002700	Keratin 7	6.7			
NM_001845	Collagen, type IV, α 1	6.3	Oxidoreductase activity		
NM_002345	Lumican	6.0	NM_020299	*AKR1B10*	134.5
AU146808	Collagen, type III, α 1	4.6	AW190565	*LOXL4*	9.3
X05610	Collagen, type IV, α 2	4.6	BC001886	*RRM2*	6.6
M80927	Chitinase 3-like 1	4.5	NM_000903	*NQO1*	6.3
N30339	Collagen, type V, α 1	4.3	AY007239	*MOXD1*	4.9
			NM_003956	*CH25H*	4.4
Chemokine activity					
NM_002993	*CXCL6*	27.7	Enzyme inhibitor activity		
NM_004591	*CCL20*	23.0	NM_003122	*SPINK1*	12.8
NM_000584	Interleukin 8	12.2	NM_020169	Latexin	5.6
S69738	*CCL2*	6.1	NM_003254	*TIMP1*	4.9
NM_001511	*CXCL1*	5.8	AL541302	*SERPINE2*	4.3
Calcium Ion binding			Other functions		
AI826799	*EFEMP1*	14.1	M83248	Osteopontin	24.7
NM_003247	Thrombospondin 2	8.9	NM_002423	*MMP7*	17.1
BF218922	Versican	7.5	NM_005764	*PDZK1IP1*	15.6
NM_005980	S100P	7.0	NM_002354	*EPCAM*	12.0
BC001388	Annexin A2	6.2	J04152	*TACSTD2*	10.9
NM_014624	*S100A6*	4.1	AW444617	*DCDC2*	10.8

Results expressed in fold change of AH vs. NASH.
