# Supplementary material for: LPS-TLR4 Pathway Mediates Ductular Cell Expansion in Alcoholic Hepatitis
Source: Sci Rep. 2016 Oct 18;6:35610. doi: 10.1038/srep35610 (PMC5067590; doi:10.1038/srep35610)
Supplement: Supplementary Information [file srep35610-s1.docx]

**SUPPLEMENTARY INFORMATION**

**Manuscript title:**

**LPS-TLR4 Pathway Mediates Ductular Cell Expansion in Alcoholic Hepatitis.**

GEMMA ODENA^1^, JIEGEN CHEN^1^, JUAN JOSE LOZANO^2^, JOSE ALTAMIRANO^2^, DANIEL RODRIGO-TORRES^2^, SILVIA AFFO^2^, ORIOL MORALES-IBANEZ^2^, HIROSHI MATSUSHITA^3^, JIAN ZOU^4^, RALUCA DUMITRU^5^, JUAN CABALLERIA^2,6^, PERE GINES^2,6^, VICENTE ARROYO^2,6^, MIN YOU^7^, PIERRE-EMMANUEL RAUTOU^8,9^, DOMINIQUE VALLA^8^, FULTON CREWS^4^, EKIHIRO SEKI^3^, PAU SANCHO-BRU^2^, RAMON BATALLER^1^**^,^**^2,4^*.

**SUPPLEMENTARY METHODS**

***Real-Time Polymerase Chain Reaction Analysis***

Quantitative PCR was used to confirm the expression of selected genes in liver human samples in experimental models and cell cultures. RNA was extracted using TRIzol reagent (Invitrogen, Life Technologies). Five hundred nanograms of total RNA were retro-transcribed and 200 ng of cDNA were then amplified (TaqMan Technology, Applied Biosystems, Life Technologies) in a final PCR volume of 10 µl using a StepOnePlus instrument (Applied Biosystems, Life Technologies). Assay-on-Demand probes and primers for the quantification of 18s, KRT23*,* EPCAM, KRT7, KRT19, COL1A1, ACTA2, SIRT1 were provided by Applied Biosystems. Results were normalized to 18s rRNA expression and gene expression values were calculated based on the ∆∆Ct method. The results were expressed as fold in relation with mean control samples.

***Cell cultures and in vitro assays***

Briefly, for the differentiation of H1 PSCs cells the following media were used: priming medium (RPMI 1640-B27 Life Technologies; 100 ng/mL Activin A and 50 ng/mL Wnt3a, Sigma-Aldrich); differentiation medium (80% KO DMEM medium, 20% KO Serum Replacement, 0.5% L-glutamine and 1% non-essential amino acids, Life Technologies; 0.1 mM ß-Mercaptoethanol and 1% DMSO, Sigma-Aldrich); maturation and maintenance medium (Leibovitz L-15 medium, 8.3% tryptose phosphate, 8.3% heat inactivated fetal bovine serum, 10 µM hydrocortisone 21-hemisuccinate, 1 µM Insulin, 1% L-glutamine, 0.2% ascorbic acid, 10 ng/mL human hepatocyte growth factor and 20 ng/mL oncostatin M, Life Technologies). BAML cells were maintained on type-I collagen-coated plates in MCDB201/DMEM medium (20% MCDB201, 70% DMEM, 10% FBS, 25 mM HEPES, 100 µM β-Mercaptoethanol, 10 mM Nicotinamide, 1 µg/ml human insulin and 100 nM dexamethasone, Sigma-Aldrich; 20 ng/ml epidermal growth factor, R&D Systems, Minneapolis, MN; and 10% FBS, Gemini Bio-Products, West Sacramento, CA).

***Analysis of Plasma free and microparticle-bound KRT23 levels***

To analyze plasma free and microparticle-bound KRT23 levels, citrated venous blood (15 mL) was centrifuged at 500 *g* for 15 minutes to remove cells as described previously ^1^. Briefly, the supernatant of the first centrifugation was then centrifuged at 15,200 *g* for 5 minutes to remove cell debris and apoptotic bodies (+18°C).  H-Gly-Pro-Arg-Pro-OH (5 μmol/L; Calbiochem, La Jolla, CA) was then added, and the samples were stored frozen at -80°C until use. To assess microparticle-bound KRT23 levels, soluble keratin 23 levels were determined using commercially available immunoassays (Cusabio) in the platelet-free plasma of controls and of patients both before and after 2 successive 0.2-μm filtrations (Ceveron MFU 500; Technoclone, Vienna, Austria). The difference between soluble keratin-23 levels in initial and in filtrated platelet-free plasma reflected was assessed.

***Studies in Liver Slices***

Liver slices represent an *in vitro* model that contains multiple cell types, structures and microenvironments similar to the whole liver. Liver slices were prepared using 5-day-old rat Sprague-Dawley rat neonates (Charles River, Raleigh, NC). The protocol was based on previous published work brain slices ^2^. Briefly, livers were dissected from 5-day-old neonates in Gey’s buffer and sectioned at 375 μm with McILwain tissue chopper (Mickle Engineering Company Ltd, Surrey, UK). Slices were transferred to a 30-mm diameter membrane tissue insert (Millicell-CM; Millipore Corp., Bedford, MA), 6 slices per membrane, placed in 6-well plates, and cultured in 1.2 ml of DMEM medium (Gibco, Life Technologies, Grand Island, NY) containing 10% fetal bovine serum (FBS) and L-glutamine (2 mM, Sigma-Aldrich). Liver slices were cultured 9 days *in vitro* before any treatment. For siRNA experiments, liver slices at 8 days *in vitro* were transfected with either negative control RNA (scramble) or specific TLR4 siRNA (Ambion, Life Technologies) for 24 h and followed by treatment with LPS (50 ng/mL, Sigma-Aldrich) for 24 h in fresh culture medium. At the end of experiment, the slices were removed for quantitative PCR analysis. All protocols followed in this study were approved by the Institutional Animal Care Use Committee and were in accordance with National Institute of Health regulation for the care and use of animal in research.

***Histochemical and Immunostaining studies***

Immunohistochemistry studies for KRT23 expression were performed in liver specimens from patients with AH (n=6), healthy liver controls (n=6) and WT and *Tlr4*-KO mice. For immunohistochemistry studies 3 μm paraffin-embedded liver sections were deparaffinized and incubated in Target Retrieval Solution, Citrate pH6 (Dako, Carpinteria, CA) heated in a pressure cooker for 20 minutes or were rehydrated and antigen retrieved with EnVision Flex Target Retrieval Solution Low pH (Dako) in a Dako PT Link. Sections were incubated with anti-KRT23 (Supplementary Table 4) overnight at 4°C and secondary antibodies for 1 hour at room temperature. Liver paraffin sections from animal models were also stained with anti-KRT23 antibody following the same protocol. In addition, a hematoxylin and eosin staining was performed following standard protocols. For immunofluorescence studies, liver specimens were cut into 5 μm thick sections and incubated in citrate buffer (pH=6) for 20 minutes before the addition of the primary antibody (Supplementary Table 4). LPC cells specimens were fixed with 4% paraformaldehyde for 30 min at 4°C and permeabilized in 0.3% Triton X-100 (Sigma-Aldrich) for 15 min then blocked in PBS containing 10% goat serum for 15 min. Both liver and LPCs samples were mounted with Mounting Medium for Fluorescence with 4',6-diamidino-2-phenylindole (DAPI) (Vector Laboratories, Burlingame, CA) to ensure nuclear staining. Immunofluorescence staining in liver samples was analyzed with a Nikon Eclypse E600. Images were processed with Cell^F software (Olympus Soft Imaging, Münster, Germany). Images for LPCs were acquired using an Olympus IX81 zero-drift microscope (Revolution XD, Andor Technology, Belfast, UK).

***Western Blotting***

Protein extracts from human LPCs differentiated in culture and from liver samples from experimental models were analyzed by western blotting. Membranes were incubated with primary antibodies (Supplementary Table 4) overnight at 4°C and secondary antibodies for 1 hour at room temperature. GAPDH was used as endogenous control. Proteins were detected by Luminata Crescendo Western HRP substrate (Millipore, Billerica, MA) and were visualized using FluorChem™ E System (ProteinSimple, San Jose, CA). The quantification of the proteins was performed by densitometric analysis using and ImageJ software (NIH National Institutes of Health, Bethesda, MD).

**SUPPLEMENTARY METHODS REFERENCES**

1 Rautou, P. E. *et al.* Abnormal plasma microparticles impair vasoconstrictor responses in patients with cirrhosis. *Gastroenterology* **143**, 166-176.e166, doi:10.1053/j.gastro.2012.03.040 (2012).

2 Zou, J. & Crews, F. Induction of innate immune gene expression cascades in brain slice cultures by ethanol: key role of NF-kappaB and proinflammatory cytokines. *Alcoholism, clinical and experimental research* **34**, 777-789, doi:10.1111/j.1530-0277.2010.01150.x (2010).

**Supplentary Table 1**. Baseline characteristics of patients with NASH.

| **Characteristics** | | **Median (SE or %)** |
| --- | --- | --- |
| Age (years) | | 47 (4) |
| Male n (%) | | 6 (60) |
| Body mass index (BMI) | | 45 (1) |
| Homeostatic Metabolic Assesment (HOMA)-index | | 12 (3) |
| **Laboratory and hemodynamic parameters** | | |
| Glucose (mg/dl) | | 123 (16) |
| Cholesterol | | 191 (10) |
| Triglycerides | | 150 (14) |
| AST ( U/L) | | 39 (5) |
| ALT (U/L) | | 52 (10) |
| GGT (U/L) | | 42 (9) |
| Serum bilirubin (mg/dL) | | 0.5 (0.03) |
| **Fibrosis Stage** | | |
| None [n (%)] | | 1 (10) |
| Perisinusoidal or periportal (1) [n (%)] | | 4 (40) |
|  | Mild, zone 3, perisinusoidal (1a) | 2 |
|  | Moderate, zone 3, perisinusoidal (1b) | 1 |
|  | Portal/periportal (1c) | 1 |
|  | Perisinusoidal and portal/periportal (2)[n (%)] | 1 (10) |
|  | Bridging fibrosis (3) [n (%)] | 4 (40) |
|  | Cirrhosis (4) [n (%)] | 1 (10) |
| **NAS Score** | | |
| Steatosis [n (%)] | |  |
| 0 (<5%) | | 1 (10) |
| 1 (5-33%) | | 1 (10) |
| 2 (34-66%) | | 6 (60) |
| 3 (>66%) | | 2 (20) |
| Lobular Inflammation [n (%)] | |  |
| 0 (none) | | 1 (10) |
| 1 (<2 foci/200x) | | 4 (20) |
| 2 (2-4 foci/ 200x) | | 4 (40) |
| 3 (>4 foci/200x) | | 1 (10) |
| Hepatocyte damage (ballooning) [n (%)] | |  |
| 0 (no) | | 0 (0) |
| 1 (few) | | 8 (80) |
| 2 (many/prominent) | | 2 (20) |
| Total score [n, (%)] | |  |
| 0-2 | | 0 (0) |
| 3-4 | | 6 (60) |
| 5-8 | | 4 (40) |

**Supplementary Table 2.** Most significant molecular function pathways for upregulated genes in patients with AH when compared to patients with NASH revealed in the functional analysis.

| **Pathways** | **GOMF ID** | **Involved genes** | **FDR** |
| --- | --- | --- | --- |
| Structural molecule activity | GO:0005198 | 78 | <0.001 |
| Identical protein binding | GO:0042802 | 89 | 0.001 |
| GTP binding | GO:0005525 | 44 | 0.001 |
| Protein C-terminus binding | GO:0008022 | 25 | 0.001 |
| Chemokine activity | GO:0008009 | 11 | 0.001 |
| Growth factor binding | GO:0019838 | 19 | 0.001 |
| Guanyl nucleotide binding | GO:0019001 | 44 | 0.002 |
| Protein complex binding | GO:0032403 | 39 | 0.005 |
| Calcium ion binding | GO:0005509 | 64 | 0.013 |
| Hydrolase activity, acting on acid anhydrides | GO:0016817 | 75 | 0.013 |
| Heparin binding | GO:0008201 | 18 | 0.017 |
| Pyrophosphatase activity | GO:0016462 | 73 | 0.018 |
| Oxidoreductase activity | GO:0016491 | 65 | 0.018 |
| Protein dimerization activity | GO:0046983 | 81 | 0.019 |
| Kinase binding | GO:0019900 | 42 | 0.019 |
| Carbohydrate derivative binding | GO:0097367 | 23 | 0.028 |
| Antioxidant activity | GO:0016209 | 10 | 0.030 |
| G-protein coupled receptor binding | GO:0001664 | 22 | 0.030 |
| Protein kinase binding | GO:0019901 | 36 | 0.037 |
| Actin binding | GO:0003779 | 35 | 0.040 |
| Electron carrier activity | GO:0009055 | 17 | 0.040 |
| Sulfur compound binding | GO:1901681 | 19 | 0.040 |
| Carbohydrate binding | GO:0030246 | 21 | 0.046 |
| Enzyme inhibitor activity | GO:0004857 | 30 | 0.046 |
| Cytokine receptor binding | GO:0005126 | 22 | 0.046 |

**Supplementary Table 3.** Most significant molecular function pathways for downregulated genes in patients with AH when compared to patients with NASH revealed in the functional analysis.

| **Pathways** | **GOMF ID** | **Involved genes** | **FDR** |
| --- | --- | --- | --- |
| Oxidoreductase activity | GO:0016491 | 99 | <0.0001 |
| Cofactor binding | GO:0048037 | 47 | <0.0001 |
| Electron carrier activity | GO:0009055 | 28 | <0.0001 |
| Coenzyme binding | GO:0050662 | 31 | <0.0001 |
| Pyridoxal phosphate binding | GO:0030170 | 15 | <0.0001 |
| Monooxygenase activity | GO:0004497 | 19 | <0.0001 |
| Iron ion binding | GO:0005506 | 25 | <0.0001 |
| Protein homodimerization activity | GO:0042803 | 55 | <0.0001 |
| Tetrapyrrole binding | GO:0046906 | 22 | <0.0001 |
| Lyase activity | GO:0016829 | 23 | <0.0001 |
| Steroid hormone receptor activity | GO:0003707 | 13 | <0.0001 |
| Heme binding | GO:0020037 | 20 | <0.0001 |
| Hydrolase activity, acting on carbon-nitrogen (but not peptide) bonds | GO:0016810 | 17 | <0.0001 |
| Carboxylic acid binding | GO:0031406 | 22 | <0.0001 |
| Identical protein binding | GO:0042802 | 68 | <0.0001 |
| NAD binding | GO:0051287 | 10 | <0.0001 |
| Carbon-oxygen lyase activity | GO:0016835 | 11 | <0.0001 |
| Ligand-activated sequence-specific DNA binding RNA polymerase II transcription factor activity | GO:0004879 | 10 | 0.0001 |
| Carbohydrate binding | GO:0030246 | 22 | 0.0002 |
| Vitamin binding | GO:0019842 | 12 | 0.0002 |
| Flavin adenine dinucleotide binding | GO:0050660 | 11 | 0.0005 |
| Amino acid binding | GO:0016597 | 13 | 0.0005 |
| Dioxygenase activity | GO:0051213 | 12 | 0.0008 |
| Organic anion transmembrane transporter activity | GO:0008514 | 14 | 0.001 |
| Carbohydrate derivative binding | GO:0097367 | 20 | 0.001 |

**Supplementary Table 4.** List of antibodies used for immunohistochemistry and immunofluorescense stainings and Western blot experiments.

| **Primary antibody** | **Species** | **Supplier** | **Catalog number** |
| --- | --- | --- | --- |
| KRT23 | Rabbit | Sigma-Aldrich | HPA012050 |
| KRT7 | Rabbit | Dako | M701801 |
| KRT19 | Rabbit | Sigma-Aldrich | HPA002465 |
| EPCAM | Mouse | Dako | M080429 |
| SIRT1 | Rabbit | Santa Cruz Biotechnology | sc-15404 |
| GAPDH | Mouse | Abcam | ab8245 |

**
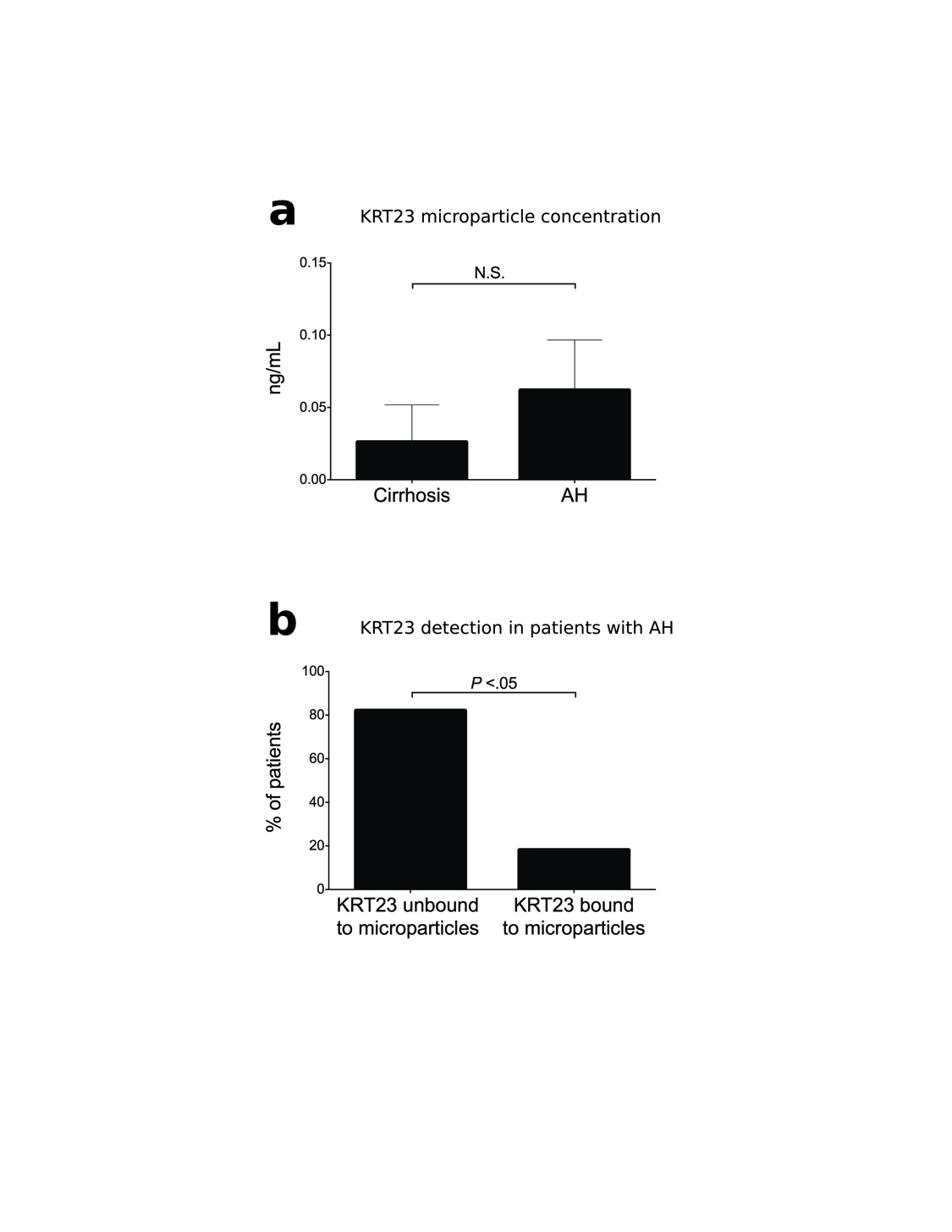
**

**Supplementary Figure 1. Microparticle-bound KRT23 levels**

**a-b.** Microparticle-bound KRT23 levels. KRT23 was mainly found unbound to microparticles in patients with AH (n=34) and in patients with cirrhosis (n=14).


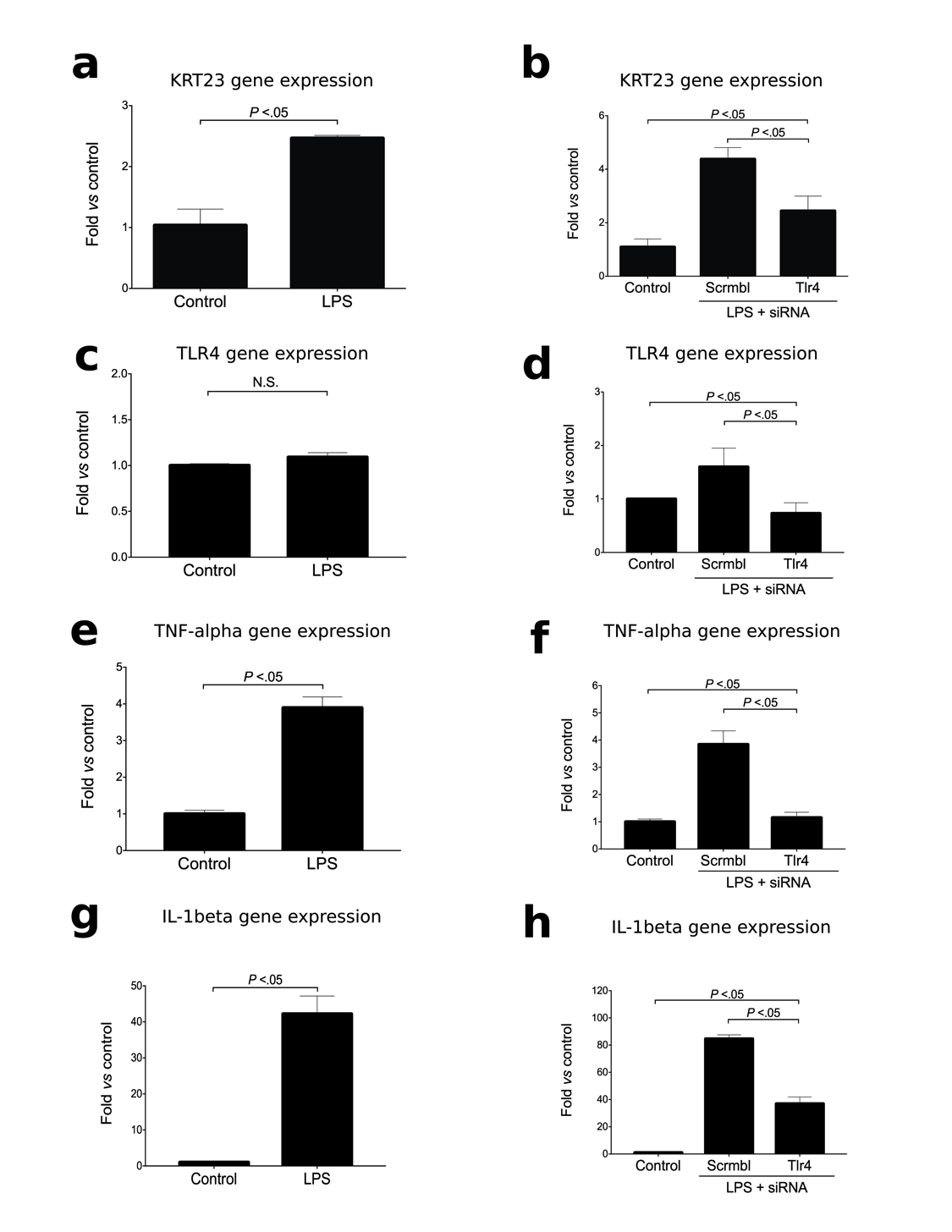


**Supplementary Figure 2. KRT23 gene expression in Liver slices**

*Krt23* gene expression was measured in precision-cut liver slices obtained from 5-day-old rat neonates. Liver slices were cultured 9 days *in vitro* and then treated with LPS (50 ng/mL) for 24 h. **a.** LPS treatment induced *Krt23* gene expression (3 independent experiments). **b.** Liver slices at 8 days *in vitro* were transfected with either negative control RNA (scramble) or specific *Tlr4* siRNA for 24 h and followed by treatment with LPS (50 ng/mL) for 24 h in fresh culture medium (3 independent experiments). *Tlr4* silencing decreased LPS-induced *Krt23* gene expression. **c.** LPS treatment did not increase *Tlr4* gene expression. **d.** *Tlr4* silencing was shown when compared to liver slices transfected with negative control RNA (scramble). **e.** LPS treatment induced *TNF-alpha* gene expression. **f.** *Tlr4* silencing decreased LPS-induced *TNF-alpha* gene expression. **g.** LPS treatment induced *IL-1beta* gene expression. **h.** *Tlr4* silencing decreased LPS-induced *IL-1beta* gene expression.

**
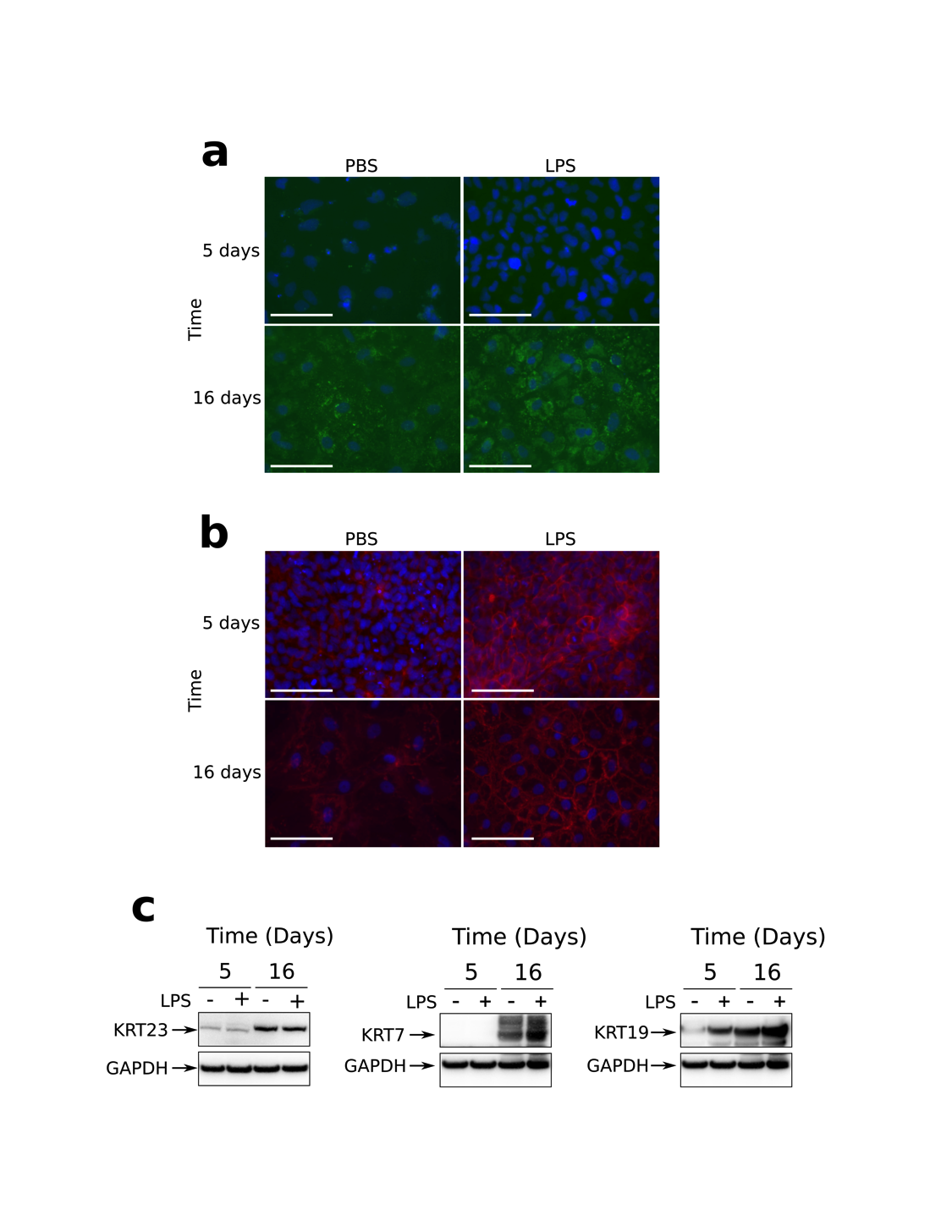
**

**Supplementary Figure 3. Effects of LPS on a cell model of LPCs differentiation**

**A.** Representative pictures of KRT23 of immunofluorescence staining (green) in LPCs generated from pluripotent stem cells**.** Nuclei counterstaining was performed with DAPI (blue) (×100 magnification). **B.** Representative pictures of EPCAM immunostaining (red) in differentiated LPCs**.** Nuclei counterstaining was performed with DAPI (blue) (×100 magnification). **C.** Representative Western blots of KRT23, KRT7 and KRT19 protein detection in a cell model of LPCs differentiation (3 independent experiments). Bars, 100 µm.

**
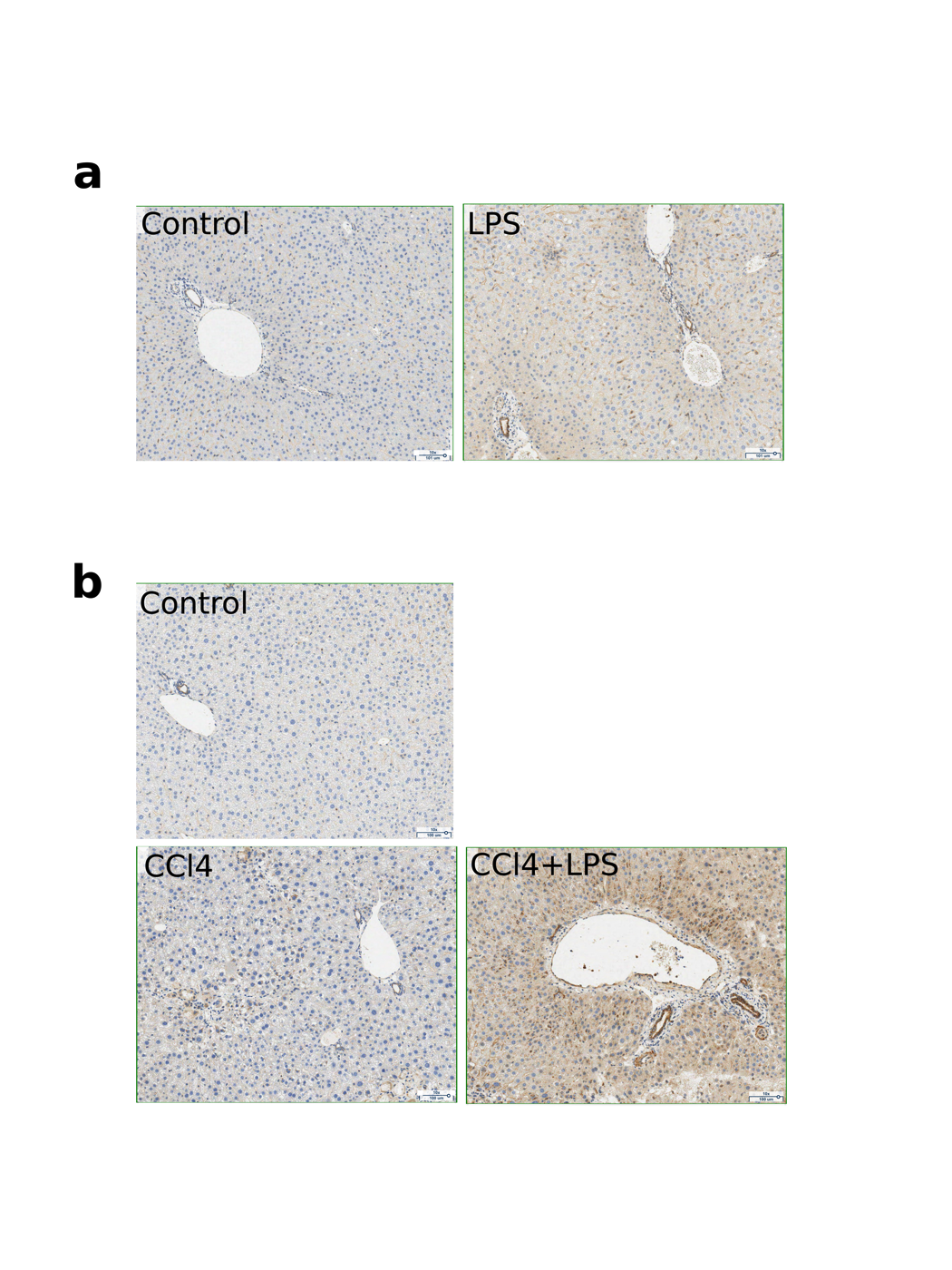
**

**Supplementary Figure 4. KRT23 protein detection in LPS mice model and LPS+CCl_4_ mice model.**

**a.** Representative pictures of KRT23 of immunohistochemistry staining in a mouse model of acute liver injury (single injection of LPS). (×10 magnification). **B.** Representative pictures of KRT23 of immunohistochemistry staining in a mouse model of advanced fibrosis (CCl4 + LPS). (×10 magnification). Bars, 100 µm.

**
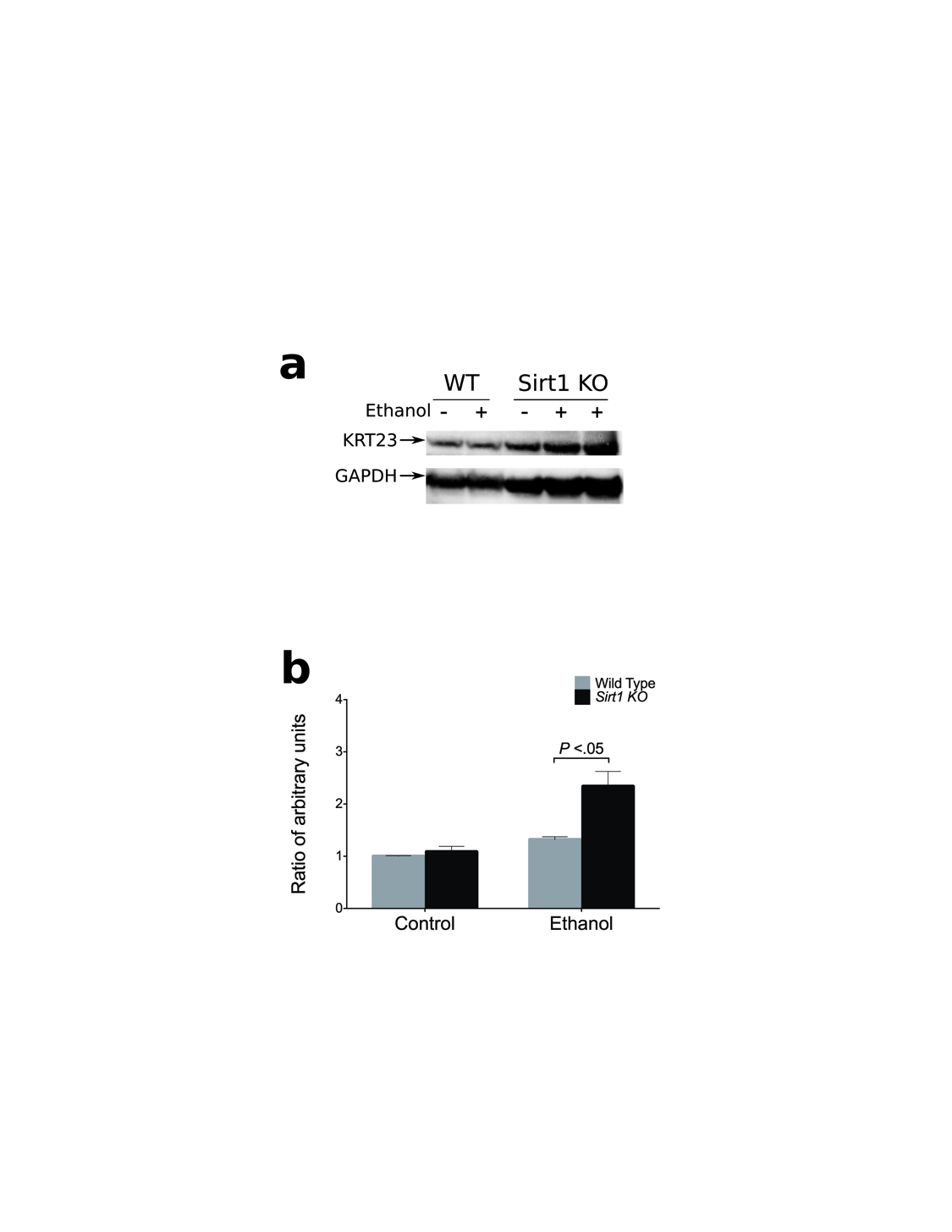
**

**Supplementary Figure 5. KRT23 protein detection in SIRT1-KO mice**

**A.** Representative Western blot of Krt23 protein detection in WT (n=6) and *Sirt1*-KO (n=6) mice subjected to a model of chronic plus single binge ethanol feeding (the NIAAA model). **B.** Krt23 protein relative quantification in WT and *Sirt1*-KO mice subjected to a model of chronic plus single binge ethanol. Krt23 is represented as mean ratio values quantified from protein bands compared to WT control mice (n=6).

**
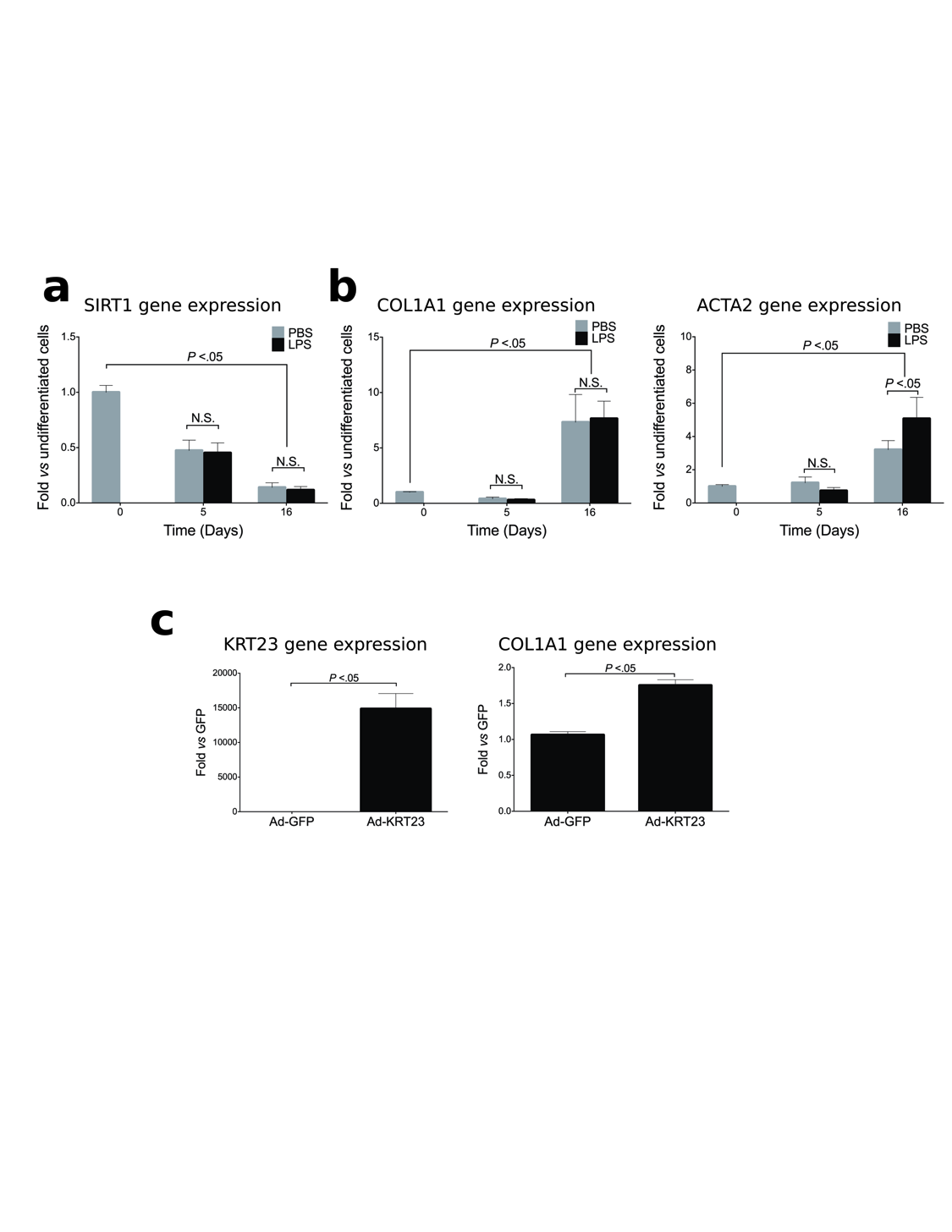
**

**Supplementary Figure 6. KRT23 regulation by HDACs in human liver progenitor cells and KRT23 effects on collagen synthesis.**

**a.** Gene expression of *Sirt1*, an HDAC, in a model of liver progenitor cells differentiation (3 independent experiments). **b.** Human LPC differentiated in culture also showed an increase in *COL1A1* expression as well as in α-smooth muscle actin (*ACTA2*). **c.** Effect of *KRT23* overexpression with adenovirus vectors containing either a specific *KRT23* sequence (Ad-*KRT23*) or a *GFP* sequence (Ad-*GFP*) in HepaRG cells (3 independent experiments). Infection of HepaRG cells with an adenovirus-encoding *KRT23* promoted *COL1A1* expression.
